# Peptide-mediated microalgae harvesting method for efficient biofuel production

**DOI:** 10.1186/s13068-015-0406-9

**Published:** 2016-01-13

**Authors:** Yoshiaki Maeda, Takuma Tateishi, Yuta Niwa, Masaki Muto, Tomoko Yoshino, David Kisailus, Tsuyoshi Tanaka

**Affiliations:** Division of Biotechnology and Life Science, Institute of Engineering, Tokyo University of Agriculture and Technology, 2-24-16, Naka-cho, Koganei, Tokyo 184-8588 Japan; JST, CREST, Sanbancho 5, Chiyoda-ku, Tokyo, 102-0075 Japan; Department of Chemical and Environmental Engineering, University of California, Riverside, Room 343 Materials Science and Engineering Building, Riverside, CA 92521 USA

**Keywords:** Biofuel production, Harvesting, Sedimentation, Frustulin, Cell-surface display, Silica-affinity peptide, Diatom, *Fistulifera solaris* JPCC DA0580

## Abstract

**Background:**

Production of biofuels from microalgae has been recognized to be a promising route for a sustainable energy supply. However, the microalgae harvesting process is a bottleneck for industrialization because it is energy intensive. Thus, by displaying interactive protein factors on the cell wall, oleaginous microalgae can acquire the auto- and controllable-flocculation function, yielding smarter and energy-efficient harvesting.

**Results:**

Towards this goal, we established a cell-surface display system using the oleaginous diatom *Fistulifera solaris* JPCC DA0580. Putative cell wall proteins, termed frustulins, were identified from the genome information using a homology search. A selected frustulin was subsequently fused with green fluorescent protein (GFP) and a diatom cell-surface display was successfully demonstrated. The antibody-binding assay further confirmed that the displayed GFP could interact with the antibody at the outermost surface of the cells. Moreover, a cell harvesting experiment was carried out using silica-affinity peptide-displaying diatom cells and silica particles where engineered cells attached to the silica particles resulting in immediate sedimentation.

**Conclusion:**

This is the first report to demonstrate the engineered peptide-mediated harvesting of oleaginous microalgae using a cell-surface display system. Flocculation efficiency based on the silica-affinity peptide-mediated cell harvesting method demonstrated a comparable performance to other flocculation strategies which use either harsh pH conditions or expensive chemical/biological flocculation agents. We propose that our peptide-mediated cell harvest method will be useful for the efficient biofuel production in the future.

**Electronic supplementary material:**

The online version of this article (doi:10.1186/s13068-015-0406-9) contains supplementary material, which is available to authorized users.

## Background

With an increased demand for a sustainable energy supply, biofuel production has attracted much attention [1–4]. Microalgal biodiesel production has been investigated to meet such demands due to its advantageous features (e.g., global carbon dioxide fixation, no competition with food sources, significantly greater biomass yield than higher plants, and oil accumulation at a high level inside the cells) [5, 6]. Several eukaryotic microalgae can accumulate triacylglycerol (TAG) in high levels as a form of oil droplets, and some promising oil producers have been intensively studied to understand the TAG biosynthesis [7, 8]. In such pursuits, we recently identified an oleaginous diatom, *Fistuliferasolaris* JPCC DA0580 from our marine microalgal culture collection [9, 10]. A beneficial feature of this diatom for biodiesel production is its high total lipid content (~60 %, w/w) [11], leading to high oil productivity [12]. Bench-scale (~200 L) outdoor mass cultivation has been studied using raceway and column bioreactors [13]. Steady growth of *F. solaris* with areal oil productivity of 3.23 g/m^2^/day was confirmed and little bacterial contamination was observed.

However, even though promising microalgal strains are used, the efficiency of energy production using microalgal biomass is not currently recognized to be sufficient. Energy production efficiency is usually expressed by the ratio of energy production to energy consumption (i.e., termed energy profit ratio). Although the current ratio derived from the microalgal biomass is desired to be more than 1 (i.e, energy production is higher than the consumption), in practice, it is difficult to achieve [14]. Towards this goal, tremendous efforts have been made to enhance TAG accumulation in target microalgal cells by means of cultivation techniques [15] as well as metabolic engineering [16]. However, researchers determined that such approaches directing enhancement of energy production are not sufficient due to the significant energy consumption in their processes. Therefore, decreasing this energy consumption during the microalgal biomass processing is critical to the practicality of microalgal biofuels.

Recently, careful investigation of process design parameters revealed that the harvesting process (i.e., collection of microalgal biomass from the liquid medium) is one of the steps responsible for a large portion of energy consumption [17, 18]. Conventional centrifugation or filtering systems cannot attain the strictly designed process goals aimed at minimizing energy consumption [18, 19]. Several alternative processes for microalgal harvesting have been proposed [19, 20]. Among them, flocculation is a possible harvesting strategy without consuming large amounts of energy. Specifically, flocculant agents (e.g., multivalent cations and cationic polymers) can readily induce aggregation by interacting with the negatively charged microalgal cell surface [19]. However, the cost of flocculants is currently an issue. Another proposed strategy is pH-responsible cell flocculation [21, 22]. However, large quantities of harsh acids or alkalis would be necessary to control the pH of culture media when mass cultivation is performed for practical production. The next challenge is to add an auto- and controllable-flocculation function to the microalgal cell surface so that a smarter harvesting process can be established. In this strategy, interactive protein factors, which result in self-aggregation and lead to bridging the cells for flocculation are attached on the surface of the microalgal cells. Using an inducible expression system [23, 24], timing the control of harvesting is also possible. To achieve this goal, it is essential to establish a technique for protein display on the surfaces of microalgae, in particular, the oleaginous diatom *F. solaris*.

Protein displays on the diatom cells have been rarely demonstrated. To date, only a non-oleaginous diatom, *Thalassiosira pseudonana*, had displayed proteins of interest in the form of fusion proteins with the native cell wall (termed frustule) proteins [25, 26]. Thus, identification of frustule-associated proteins as anchor molecules is imperative for the success of diatom-cell-surface display technologies. However, there are only a few diatoms whose frustule-associated proteins are well studied. Frustule-associated proteins are species-specific, and thus the challenge is that they need to be identified from individual diatoms prior to establishment of the display system.

In this study, we identified frustule-associated proteins, termed frustulins, from the genome of the oleaginous diatom, *F. solaris* [27]. A selected frustulin was evaluated as an anchor for protein display by fusing with green fluorescent protein (GFP), and the protein display efficiency was compared to that using the different frustule-associated protein identified in our previous study [28]. Furthermore, cell harvesting experiments were performed to demonstrate whether displayed protein-mediated harvesting is possible, in which a silica-affinity peptide (SAP) was displayed on the diatom cells, and silica particles were added to induce flocculation. The engineered peptide-mediated smart harvesting strategy established in this study should be useful in efficient biofuel production.

## Results and discussion

### Primary structure and gene expression level of frustulins of *F. solaris*

**Fig. 1:**
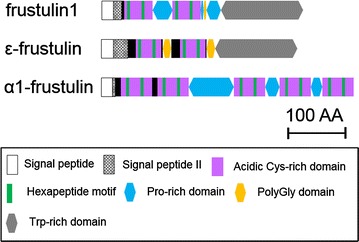
Schematic primary structures of frustulins. Frustulins 1 (formerly G20405) found in the genome of *F. solaris* JPCC DA0580, *ε*-frustulin of *N. pelliculosa* and *α*1-frustulin of *C. fusiformis* are presented

In the present study, putative frustulin-like proteins were screened from *F. solaris* JPCC DA0580 genome using *α*1-frustulin of *Cylindrothecafusiformis* [29] and *ε*-frustulin of *Navicula pelliculosa* [30] as the query sequences, whereas our previous study had utilized only α1-frustulin for the homology search, and resulted in revealing seven putative proteins [28]. As a result of the present homology search, 15 proteins exhibited sequence similarities to these queries. However, four proteins lack the signal peptide II [30], which is a typical pre-sequence of frustule-associated proteins and responsible for directing the polypeptide into silica deposition vesicle and were thus excluded. The remaining 11 proteins met the sequence criteria of frustulins (see “Methods” section), and thus, were referred to as members of the frustulin family (Fig. 1 and Additional file 1: Fig. S1). The clustering analysis revealed that all of the selected 11 frustulins (assigned from frustulin1–frustulin11) were closely related to *ε*-frustulin of *N. pelliculosa* rather than *α*1-frustulin of *C. fusiformis* (Additional file 1: Fig. S2). This is reasonable because *F. solaris* is phylogenetically close to *N. pelliculosa* [9]. The *ε*-frustulin consists of a pre-sequence, acidic cysteine-rich (ACR) domains containing two hexapeptide motifs (i.e., CQGDCD and VPGCSG), polyglycine domains, and a tryptophan-rich domain [30]. The frustulins found in *F. solaris* JPCC DA0580 share some typical features of the pre-sequence, ACR domains, and a tryptophan-rich domain (indeed the tryptophan content is as low as 4 %, Additional file 1: Fig. S3), but some *F. solaris*-specific features were also observed as described below. The cysteine residues in the hexapeptide motifs, which are considered to have calcium-binding properties of frustulin [29, 30], are well conserved between *ε*-frustulin and *F. solaris*-frustulins, while other amino acids in the motif are substitutable. In addition, some of *F. solaris*-frustulins do not have polyglycine domains between the ACR domains. Instead, the ACR domains are connected by proline-rich domains, which are found in *α*-frustulins rather than *ε*-frustulin. Note that frustulin3, 4, and 9 have neither polyglycine domains nor proline-rich domains (Additional file 1: Fig. S1). It could suggest that the two linker domains might not be essential for a frustulin function. When the functional motifs were explored from the frustulin sequences using an InterProScan 5 program, a ricin-type *β*-trefoil lectin domain (i.e., ricin B lectin domain, InterPro entry IPR000772) was found at individual tryptophan-rich domains, while its function still remains obscure.

To confirm the gene expression level of the frustulin proteins, reads per killobases per million (RPKM) value, acquired in our transcriptome analysis [31], were compared (Additional file 1: Table S1). During the cell growth, frustulin1, 2, 5, and 8 were substantially expressed (all RPKM values >10). Among them, frustulin1 demonstrated high RPKM values without significant fluctuation. Based on this, we employed frustulin1 as an anchor molecule for diatom cell-surface display applications.

### Diatom cell-surface display

**Fig. 2:**
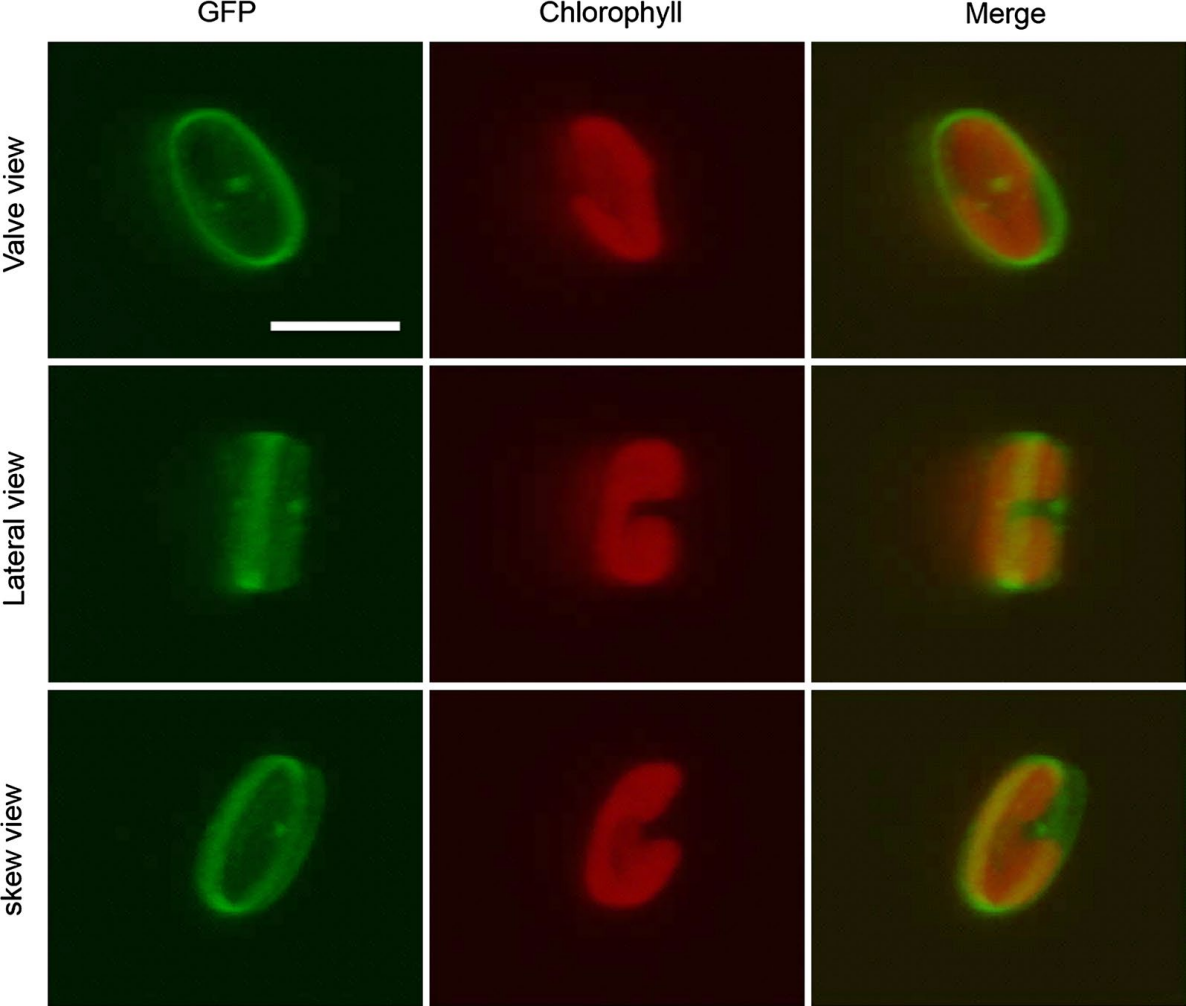
Confocal microscopic images of *F. solaris* JPCC DA0580 transformant expressing frustulin1-GFP. *Scale bar* 5 μm

To evaluate frustulin1 as a molecular anchor of the cell-surface display system, the recombinant frustulin1-GFP fusion protein was designed and expressed in *F. solaris* JPCC DA0580. Prior to observation, the fusion protein expression was confirmed with Western blotting using an anti-GFP antibody (Additional file 1: Fig. S4). Subsequently, confocal microscopy of the resulting transformant cells revealed the substantial GFP expression surrounding the cells (Fig. 2). The lateral view of the re-constructed 3D image demonstrated intensive fluorescence around the central region of the girdle face. Meanwhile, a slightly weaker fluorescence was also observed in the valve regions. When fluorescent intensity was profiled across the girdle face, the central region exhibited approximately double the intensity than the valve regions (Additional file 1: Fig. S5a–d). A similar tendency was reproducibly observed in multiple cells. The uneven distribution of fluorescence derived from frustulin1-GFP at the central part of the girdle face may be due to the structural features of the diatom frustule. Two parts of cell wall, the large epitheca and small hypotheca, face each other, and each theca overlaps at the center of the girdle face [29, 30]. This structure was also directly found in the *F. solaris* cells [9, 28]. If the frustulin1 is evenly distributed on the cell wall, the overlapped region would emit a stronger GFP fluorescence (as observed here). This is consistent with a previous report where an even distribution of frustulin on the whole surface of the cell wall of another diatom (*C. fusiformis*) was demonstrated by staining the cell using fluorescein isothiocyanate (FITC)-labeled anti-frustulin antibody [32]. This feature could be beneficial for harvesting because frustulin1 can be expected to display interactive proteins on the whole surface of the diatom cell, and consequently mediate better flocculation rather than localizing in a specific small area. The fluorescence signal from the wild-type diatoms was undetectable under the same observed conditions. Interestingly, some transformant cells had the isolated dots which emitted significant fluorescence at the valve face (Additional file 1: Fig. S5e). However, the precise localization and its function needs to be determined.

**Fig. 3:**
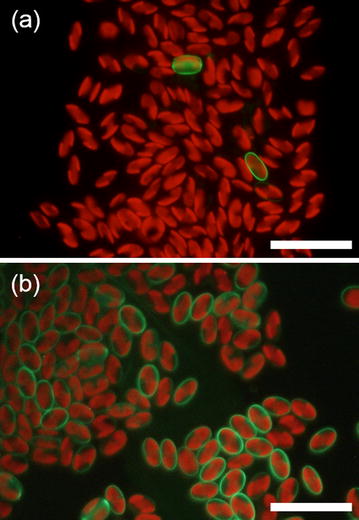
Fluorescent images of transformant cells harboring the expression genes of G7408-GFP **a** or frustulin 1-GFP **b**. *Scale bar* 20 μm

Further characterization of frustulin1 was performed to evaluate the potential of this protein as an anchor for protein display on the diatom cell. First, the expression efficiency was observed and compared to the other frustule-associated protein (i.e., G7408) identified in a previous study [28]. G7408 had been previously demonstrated to localize at the frustule. However, the transformant cells harboring the G7408-GFP expression gene readily lost the expression capacity only after a few subculturing rounds (Fig. 3a), perhaps due to the native gene silencing. In contrast, all of the transformant cells harboring the frustulin1-GFP expression gene maintained fluorescence even after repeating the subculturing (Fig. 3b). This result clearly indicates that frustulin1 is more suitable for surface protein-mediated harvesting because, in principle, only the cell displaying the engineered protein can be collected.

**Fig. 4:**
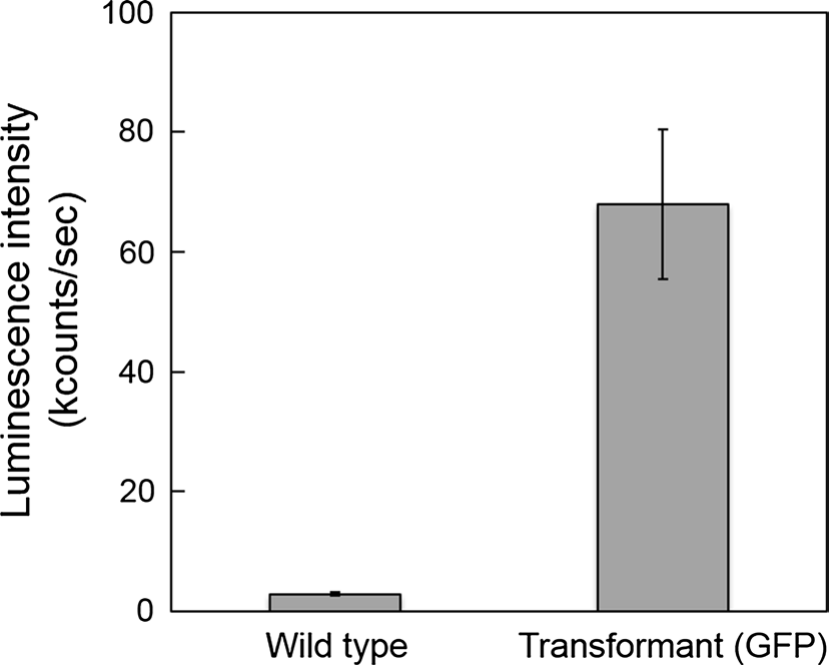
Antibody-binding assay to *F. solaris* wild-type and transformant expressing frustulin1-GFP. An alkaline phosphatase (ALP)-conjugated anti-GFP antibody was used. The luminescent substrate was added, and the ALP reacted with it to generate luminescence. *Error bars* standard deviation (*n* = 3)

To achieve surface protein-mediated harvesting of microalgal cells, it is essential for the interactive proteins to not only localize at the cell wall but also to be exposed to the outermost surface of the cell, otherwise they cannot induce flocculation. Fluorescence localization of frusutlin1-GFP at the cell wall is of not sufficient evidence of its exposure because it is well known that the extracellular polymeric substances exist at the outside of the diatom cell wall [33], beneath which frustulin1-GFP might be embedded. To confirm this, an antibody-binding assay was performed. When the antibody was added to the intact transformant cells, the binding signal was obviously higher than that from the wild-type cells (Fig. 4). This result indicated that the frustulin1-GFP was displayed on the outermost surface of the diatom cell to which the antibody proteins can be directly accessible.

The data described above enable potential cell harvesting via a diatom cell-surface display system which uses frustulin1 as an anchor molecule. To the best of our knowledge, this is the first report of a cell-surface display system for the oleaginous diatom toward the efficient bioenergy production process.

### Silica-affinity peptide-mediated cell harvesting using silica particles

**Fig. 5:**
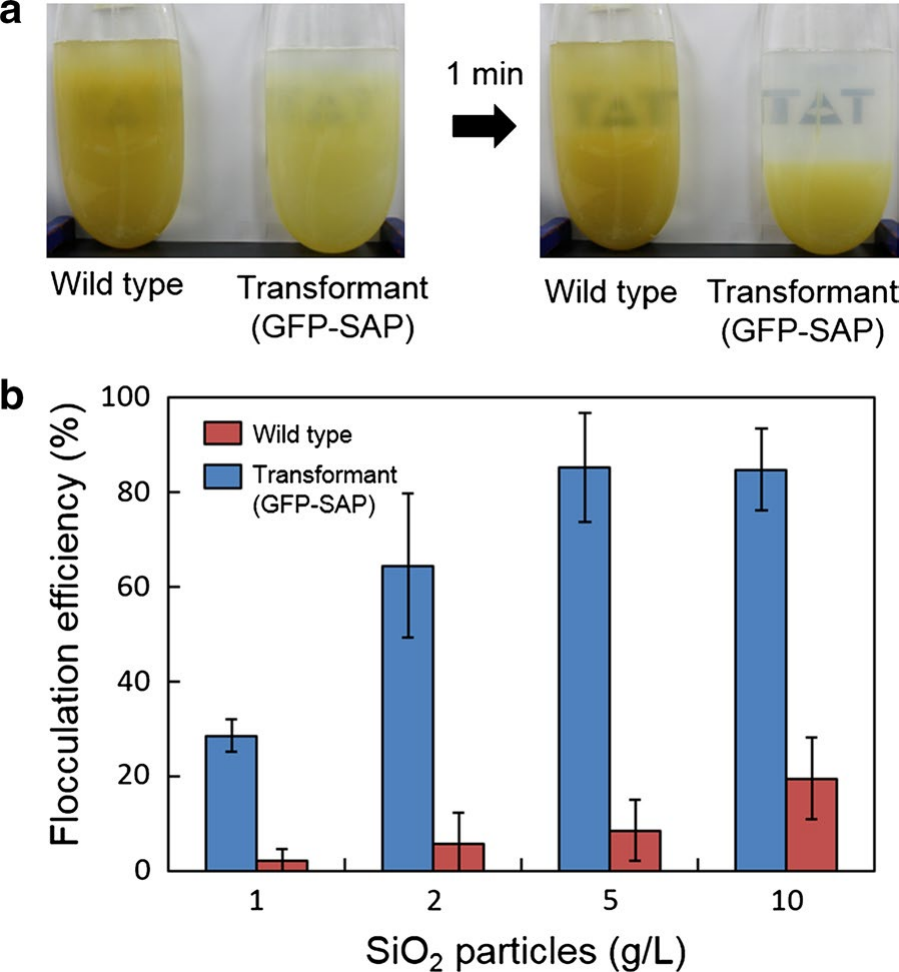
Silica-affinity peptide (SAP)-mediated cell harvesting for the *F. solaris* transformant expressing frustulin1-GFP-(G_4_S)_3_-SAP. **a** Photographs of culture media of the wild-type (left) and transformant displaying GFP-SAP (right) co-incubated with silica particles before and after flocculation. Co-flocculation of diatom transformant cells and silica particles (2.5 g) were observed within 1 min. **b** Flocculation efficiency of the transformant (*blue bars*) and wild-type (*red bars*) with silica particles. Diatom cells were co-cultivated with different concentrations of silica particles in 500 ml cultures. Culture media containing cells and particles were collected at 5 cm below the water surface before and after flocculation, and flocculation efficiency was calculated by Eq. 1. *Error bars* standard deviation (*n* = 3)

As a proof of concept, we investigated whether it is possible for the diatom cell-surface display system established here to mediate algal biomass harvesting. In this study, a silica-affinity peptide (SAP; MSPHPHPRHHHT) [34] was displayed on the diatom cell surface. SAP was fused at the C-terminus of frustulin1-GFP via a (G_4_S)_3_ peptide linker; i.e., the fusion protein was expressed as a form of frustulin1-GFP-(G_4_S)_3_-SAP, in which GFP allowed us to confirm the successful display by fluorescent observation (Supplementary Fig. S6a). The transformant cells displaying GFP-SAP were co-cultivated with the silica particles. No sedimentation was observed during the co-cultivation due to aeration that contributed to the supply of CO_2_ for photosynthesis and agitation of the culture. Once the aeration stopped, silica particles immediately precipitated within 1 min, and simultaneously, the culture medium turned transparent, indicating the precipitation of the transformant cells with the particles (Fig. 5a, see also Additional file 2: Movie S1). In contrast, little precipitation was observed when wild-type cells were co-cultivated with the silica particles (Fig. 5a). Microscopic observation of the precipitated silica particles revealed that a substantial number of the transformant cells were associated with the precipitated particles, while significantly less adhesion to wild-type cells was confirmed (Additional file 1: Fig. S6b). This result indicates that the SAP display is responsible for cell adhesion to the silica particles, and subsequently leads to cell flocculation. When, instead of co-cultivation, silica particles were added after completing the diatom cultivation, little flocculation was observed (Additional file 1: Fig. S6c). This could be because silica particles precipitated too rapidly or had limited interaction with the cells versus the time with which they aggregated. Thus, it was hard to enable sufficient interaction with the particles and all of the diatom cells. This issue might be addressable by changing the mixing condition of the particles and cells. In this co-flocculation system, it is likely that the silica particles, which carry a negative charge in f/2 medium (pH ~7), might electrostatically interact with transformant cells displaying SAP (MSPHPHPRHHHT, theoretical pI = 9.59), which have a significant fraction of basic amino acid residues. Therefore, the silica particles likely form bridges between cells, effectively increasing their mass and enabling facile sedimentation. However, details of the interaction mechanisms have yet to be determined. Flocculation efficiency was increased by increasing the amount of silica particles in cultures, reaching 85 % when 5 or 10 g/L of silica particles was added transformant culture (Fig. 5b). This rate is comparable to other flocculation studies [19, 22, 35]. When silica particles were not supplied, no flocculation occurred with both the transformant and wild-type cells, indicating that silica particles are necessary for cell harvesting. It should be noted that the transformant displaying GFP-SAP exhibited steady cell growth and oil accumulation as much as the wild-type system (Additional file 1: Fig. S7). Lipid extraction from the transformant cells was readily achieved as is the case with wild-type cells. These results indicate that the cell-surface display system established in this study does not have a significantly negative impact on the biomass and lipid productivity, as well as downstream processes after harvesting.

A striking feature of this silica-inducible and SAP-mediated cell flocculation is not to regulate the component of growth medium. Silica particles do not significantly affect the components of the medium because the f/2 medium contains sodium silicate and it inherently generates silica precipitates. This feature is beneficial, in particular, for repeated batch cultures, a culture technique for enhanced biomass production, and repeatedly utilizes a part of the culture medium after cell harvesting for subsequent batch cultures [13]. After SAP-mediated harvesting, the remaining culture components are available for the repeated batch cultures without modification. In contrast, other previously proposed flocculation methodologies (e.g., pH change [21, 22], supplemental chemical [19] and biological [35, 36] flocculation agents) drastically altered the properties of culture media, and neutralization of those impacts needs additional harsh chemicals or separation steps, both of which add cost. Our harvesting strategy does not require such additional chemicals or processes. Additionally, silica particles are inexpensive as compared with other flocculation-inducing salts and polymers such as ferric chloride, aluminum sulfate, ferric sulfate, chitosan, and polyacrylamide [19]. Comparable doses of these chemical reagents are needed for flocculation of microalgal biomass. After harvesting the diatom cells, lipid extraction is performed by adding an organic solvent (e.g., *n*-hexane). Even if silica particles were not removed from the harvested biomass, hexane extraction would not be inhibited. Therefore, we propose that this is more efficient, environmentally benign, and an inexpensive method for biomass harvesting than conventional flocculation methodologies.

Besides diatoms, cell-surface display systems were established for cyanobacteria [37–39]. Although systems for green algae have not yet been thoroughly investigated, cell wall proteins, which can serve as anchors in a cell-surface system, were well studied [40]. These studies suggest that the harvesting strategy proposed in this study could be available for a broad range of microalgae producing useful materials.

## Conclusions

Using a newly identified frustulin as an anchor molecule, we successfully demonstrated GFP display on the outermost surface of the oleaginous diatom, *F. solaris* JPCC DA0580. An affinity peptide, SAP, was displayed on the cells by fusing with the frustulin, and effective flocculation was demonstrated by co-cultivating with silica particles. Our initial trial showed considerable promise for biomass harvesting. The next challenge would be implementation of this system into large-scale bioreactors used in outdoor mass cultivation. The feasibility of this harvesting method should be statistically evaluated in large-scale outdoor cultivation systems prior to practical realization, which we will investigate in the near future. We propose that the peptide-mediated microalgal harvesting strategy established in this study will pave the way towards future highly efficient biofuel production.

## Methods

### Strain and culture conditions

The marine pennate diatom *F. solaris* JPCC DA0580 cells were maintained in f/2 medium [41] dissolved in artificial seawater. The transformed cells were cultivated in the same medium with the addition of antibiotics G-418 (500 μg/ml). Cultures (500 ml of f/2 medium in flat-shaped flasks) were incubated at 25 °C under continuous, cool-white fluorescent lights at 140 μmol/m^2^/s with aeration (0.8 l of air containing 2 % CO_2_/l/min). Genes were cloned in *Escherichia coli* TOP10 (Life Technologies, Tokyo, Japan) cultured in a lysogeny broth (LB) medium containing 50 μg/ml ampicillin at 37 °C.

### Homology search of frustulin

Two known frustulin genes, α1-frustulin derived from *C. fusiformis* [29] and *ε*-frustulin from *N. pelliculosa* [30] were used as query sequences for protein basic local alignment search tools (Protein BLAST) against the draft genome sequence of *F. solaris* JPCC DA0580 [27]. The E-value cut off was set to 10^−7^. Subsequently, it was confirmed whether the amino acid sequences of the putative frustulins meet the criteria that frustulin possesses at least three of five sequence features: (1) a pre-sequence domain which contains an endoplasmic reticulum (ER) signal peptide and another signal peptide (termed SPII) aimed at targeting the silica deposition vesicle, (2) acidic cysteine-rich (ACR) domains, (3) proline-rich domains, (4) polyglycine domains, and (5) a tryptophan-rich domain previously described [30].

### Plasmid construction and transformation

An expression vector for frustulin1 (formerly G20405, DDBJ/EMBL/GenBank database accession number AB854058) fused with GFP was constructed using general gene fusion techniques [42, 43]. We used the plasmid pSP-GFP/GAPDH containing an enhanced green fluorescence protein (GFP) gene, a glyceraldehyde 3-phosphate dehydrogenase (GAPDH) gene promoter and a fucoxanthin chlorophyll a/c-binding protein A fucoxanthin chlorophyll a/c-binding protein A (fcpA) terminator, in which an *Eco*RI site exists between the promoter and GFP gene [44]. First, the *Eco*RI site was changed to the *Eco*RV site using a Strategene Quik Change II XL site-Directed Mutagenesis Kit (Agilent Technologies, Santa Clara, CA, USA) and a primer pair (5′-CAA ACA ATC ATC GAT ATC ATG GTG AGC AAG GG-3′ and 5′-CCT TGC TCA CCA TGA TAT CGA TGA TTG TTT GTT G-3′). The modified pSP-GFP/GAPDH was then digested by *Eco*RV. The frustulin1 gene was amplified with PCR using a cDNA library prepared from *F. solaris*. Total RNA was extracted from *F. solaris* using a Concert Plant RNA Reagent (Life Technologies, Tokyo, Japan) and purified with RNeasy Mini Kit (Qiagen, Hilden, Germany). After quantification of total RNA using an Agilent RNA 6000 Nano Assay Kit (Agilent Technologies, Santa Clara, CA, USA), cDNA was obtained with a SMARTer™ RACE cDNA Amplification Kit (Takara Bio, Otsu, Japan) from 1 μg of total RNA as the template. PCR amplification was performed using the specific primer set for fso:g20405 (5′-ATG ACA GTC CTT CAG CTT TTA CTC AAG GTC-3′and 5′-GTA TTT GTT CCA GTA CGA TGT GTT GCT GC-3′). The PCR product was ligated with the modified pSP-GFP/GAPDH. For the flocculation experiment, a (G_4_S)_3_ linker and silica-affinity peptide (SAP, MSPHPHPRHHHT) [34] were added at the C-terminus of frustulin-GFP. The constructed plasmids were introduced into the *F. solaris,* respectively, by microparticle bombardment using the Biolistic PDS-1000/He Particle Delivery System (Bio-Rad Laboratories, Inc., Hercules, CA, USA) as described previously [45].

### SDS-PAGE and Western blotting

*F. solaris* (5 × 10^7^ cells) were collected by centrifugation, suspended in 100 μL of 1 % (w/v) sodium dodecyl sulfate (SDS) in aqueous solution, and boiled for 10 min. After centrifugation, supernatant was collected, and SDS sample buffer was added (final concentration of 62.5 mM Tris–HCl, pH 6.8, 5 % 2-mercaptoethanol, 2 % SDS, 5 % sucrose, and 0.002 % bromophenol blue). Denatured proteins were separated by SDS–polyacrylamide gel electrophoresis sodium dodecyl sulfate–polyacrylamide gel electrophoresis (SDS-PAGE) using a 10 % (w/v) gel. Bio-Safe Coomassie G-250 Stain (Bio-Rad laboratories, Inc., Hercules, CA, USA) was used for gel staining. For Western blotting, the separated proteins were transferred to a polyvinylidene difluoride membrane. GFP was then detected using alkaline phosphatase (ALP)-labeled anti-GFP antibody (Rockland Immunochemicals Inc., Gilbertsville, PA, USA, 1/5000 dilution from stock in PBS containing 0.05 % Tween 20). BCIP/NBT-Blue (Sigma, St. Louis, MO, USA) was used as the ALP substrate for visualization.

### Fluorescence microscopy

Transformant cells were observed using a fluorescent microscope BX51 (Olympus Corporation, Tokyo, Japan) with a U-MGFPPHQ filter set for GFP and a U-MWIG2 filter set for chlorophyll. Confocal microscopy was performed using Fluoview FV1000 (Olympus Corporation, Tokyo, Japan) with an Alexa Fluor 488 filter set for GFP and an Alexa Fluor 568 set for chlorophyll fluorescence. The images were obtained every 0.2 μm along the *z-axis* to re-construct three-dimensional (3D) images.

### Antibody-binding assay

*F. solaris* JPCC DA0580 wild-type and flustulin1-GFP expressing transformant cells (5.0 × 10^6^ cells) were centrifuged at 8500*g* for 5 min, and re-suspended in 50 μL of ALP-conjugated anti-GFP antibody (1 μg/ml, Rockland, Gilbertsville, USA) solution diluted in Tris-buffered saline containing 0.05 % tween 20 (TBST) and incubated for 30 min. The cells were then washed three times with TBST and mixed with 50 μL of Lumi-Phos^®^ 530 (Wako Pure Chemical Industries, Osaka, Japan). After 5 min, luminescence intensity was measured using a microplate reader SH-9000 (Corona electric Co., Ltd., Ibaraki, Japan).

### Silica-affinity peptide (SAP)-mediated harvesting

SAP-mediated cell harvesting experiments were performed using polydisperse silica particles (approximately 1–20 μm) that are purchased from Wako Pure Chemical Industries (Osaka, Japan). *F. solaris* wild-type and the transformant displaying GFP-SAP (5.0 × 10^5^ cells/ml as initial cell concentration) were incubated for 6 days in the absence or presence (1, 2, 5 and 10 g/l) of silica particles at 25 °C under continuous, cool-white fluorescent lights at 140 μmol/m^2^/s with aeration (0.8 l of air containing 2 % CO_2_/l/min). Subsequently, the flat-shaped flasks (stand perpendicularly) containing the cells and silica particles were vigorously shook by hands. The flocculation process was recorded using a video camera. To evaluate the flocculation efficiency, cell- and particle-suspensions were collected before and after flocculation using a pipette where the pipet tips reached were placed 5 cm below the water surface. The flocculation efficiency was calculated by following (Eq. 1).

1 $${\text{Flocculation efficiency }}\left( \% \right)\,\, = \,\,\left( {1-{A /B}} \right) \, \left( \% \right)$$

where *A* and *B* are cell concentrations after and before flocculation, respectively. Cell concentrations were determined by cell counting using a microscope and hemocytometer.

## Additional files

10.1186/s13068-015-0406-9 Supplementary Figures S1, S2, S3, S4, S5, S6, and S7. Supplementary Table S1.
10.1186/s13068-015-0406-9 Silica-affinity peptide (SAP)-mediated cell harvesting for the *F. solaris* transformant expressing frustulin1-GFP-(G_4_S)_3_-SAP. Culture media of wild type (*left*) and SAP-displaying transformant (*right*) co-incubated with silica particles (2.5 g). Co-flocculation of diatom transformant cells and silica particles were monitored.

## Abbreviations

GFP: green fluorescent protein
TAG: triacylglycerol
ACR: acidic cysteine-rich
RPKM: reads per kilobases per million
FITC: fluorescein isothiocyanate
SAP: silica-affinity peptide
LB medium: lysogeny broth medium
ER: endoplasmic reticulum
GAPDH: glyceraldehyde 3-phosphate dehydrogenase
fcpA: fucoxanthin chlorophyll a/c-binding protein A
SDS-PAGE: sodium dodecyl sulfate–polyacrylamide gel electrophoresis
ALP: alkaline phosphatase

## References

[1] Brethauer S, Wyman CE (2010). Review: continuous hydrolysis and fermentation for cellulosic ethanol production. Bioresour Technol.

[2] Chisti Y (2007). Biodiesel from microalgae. Biotechnol Adv.

[3] Show K, Lee D, Tay J, Lin C, Chang J (2012). Biohydrogen production: current perspectives and the way forward. Int J Hydrogen Energy.

[4] Wyman CE (1994). Alternative fuels from biomass and their impact on carbon dioxide accumulation. Appl Biochem Biotechnol.

[5] Smith VH, Sturm BS, Denoyelles FJ, Billings SA (2010). The ecology of algal biodiesel production. Trends Ecol Evol.

[6] Ho SH, Ye X, Hasunuma T, Chang JS, Kondo A (2014). Perspectives on engineering strategies for improving biofuel production from microalgae—a critical review. Biotechnol Adv.

[7] Matsunaga T, Matsumoto M, Maeda Y, Sugiyama H, Sato R, Tanaka T (2009). Characterization of marine microalga, *Scenedesmus* sp. strain JPCC GA0024 toward biofuel production. Biotechnol Lett.

[8] Rodolfi L, Chini Zittelli G, Bassi N, Padovani G, Biondi N, Bonini G (2009). Microalgae for oil: strain selection, induction of lipid synthesis and outdoor mass cultivation in a low-cost photobioreactor. Biotechnol Bioeng.

[9] Matsumoto M, Mayama S, Nemoto M, Fukuda Y, Muto M, Yoshino T (2014). Morphological and molecular phylogenetic analysis of the high triglyceride-producing marine diatom, *Fistulifera solaris* sp. nov. (Bacillariophyceae). Phycol Res.

[10] Matsumoto M, Sugiyama H, Maeda Y, Sato R, Tanaka T, Matsunaga T (2010). Marine diatom, *Navicula* sp. strain JPCC DA0580 and marine green alga, *Chlorella* sp. strain NKG400014 as potential sources for biodiesel production. Appl Biochem Biotechnol.

[11] Liang Y, Maeda Y, Yoshino T, Matsumoto M, Tanaka T (2014). Profiling of fatty acid methyl esters from the oleaginous diatom *Fistulifera* sp. strain JPCC DA0580 under nutrition-sufficient and -deficient conditions. J Appl Phycol.

[12] Satoh A, Ichii K, Matsumoto M, Kubota C, Nemoto M, Tanaka M (2013). A process design and productivity evaluation for oil production by indoor mass cultivation of a marine diatom, *Fistulifera* sp. JPCC DA0580. Bioresour Technol.

[13] Sato R, Maeda Y, Yoshino T, Tanaka T, Matsumoto M (2014). Seasonal variation of biomass and oil production of the oleaginous diatom *Fistulifera* sp. in outdoor vertical bubble column and raceway-type bioreactors. J Biosci Bioeng.

[14] Razon LF, Tan RR (2011). Net energy analysis of the production of biodiesel and biogas from the microalgae: *haematococcus pluvialis* and *Nannochloropsis*. Appl Energy.

[15] Gardner R, Peters P, Peyton B, Cooksey KE (2011). Medium pH and nitrate concentration effects on accumulation of triacylglycerol in two members of the *Chlorophyta*. J Appl Phycol.

[16] Trentacoste EM, Shrestha RP, Smith SR, Gle C, Hartmann AC, Hildebrand M (2013). Metabolic engineering of lipid catabolism increases microalgal lipid accumulation without compromising growth. Proc Natl Acad Sci.

[17] Sander K, Murthy GS (2010). Life cycle analysis of algae biodiesel. Int J Life Cycle Assess.

[18] Uduman N, Qi Y, Danquah MK, Forde GM, Hoadley A (2010). Dewatering of microalgal cultures: a major bottleneck to algae-based fuels. J Renew Sustain Energy.

[19] Molina Grima E, Belarbi EH, Acien Fernandez FG, Robles Medina A, Chisti Y (2003). Recovery of microalgal biomass and metabolites: process options and economics. Biotechnol Adv.

[20] Pragya N, Pandey KK, Sahoo P (2013). A review on harvesting, oil extraction and biofuels production technologies from microalgae. Renew Sustain Energy Rev.

[21] Horiuchi J, Ohba I, Tada K, Kobayashi M, Kanno T, Kishimoto M (2003). Effective cell harvesting of the halotolerant microalga *Dunaliella tertiolecta* with pH control. J Biosci Bioeng.

[22] Liu J, Zhu Y, Tao Y, Zhang Y, Li A, Li T (2013). Freshwater microalgae harvested via flocculation induced by pH decrease. Biotechnol Biofuels.

[23] Poulsen N, Chesley PM, Kroger N (2006). Molecular genetic manipulation of the diatom *Thalassiosira pseudonana* (Bacillariophyceae). J Phycol.

[24] Poulsen N, Kroger N (2005). A new molecular tool for transgenic diatoms: control of mRNA and protein biosynthesis by an inducible promoter-terminator cassette. FEBS J.

[25] Poulsen N, Berne C, Spain J, Kroeger N (2007). Silica immobilization of an enzyme through genetic engineering of the diatom *Thalassiosira pseudonana*. Angew Chem Int Ed Engl.

[26] Marshall KE, Robinson EW, Hengel SM, Pasa-Tolic L, Roesijadi G (2012). FRET imaging of diatoms expressing a biosilica-localized ribose sensor. PLoS ONE.

[27] Tanaka T, Maeda Y, Veluchamy A, Tanaka M, Abida H, Marechal E (2015). Oil accumulation by the oleaginous diatom *Fistulifera solaris* as revealed by the genome and transcriptome. Plant Cell.

[28] Nemoto M, Maeda Y, Muto M, Tanaka M, Yoshino T, Mayama S (2014). Identification of a frustule-associated protein of the marine pennate diatom *Fistulifera* sp. strain JPCC DA0580. Mar Genomics.

[29] Kroger N, Bergsdorf C, Sumper M (1994). A new calcium binding glycoprotein family constitutes a major diatom cell wall component. EMBO J.

[30] Kroger N, Bergsdorf C, Sumper M (1996). Frustulins: domain conservation in a protein family associated with diatom cell walls. Eur J Biochem.

[31] Liang Y, Maeda Y, Sunaga Y, Muto M, Matsumoto M, Yoshino T (2013). Biosynthesis of polyunsaturated fatty acids in the oleaginous marine diatom *Fistulifera* sp. strain JPCC DA0580. Mar Drugs.

[32] Kroger N, Lehmann G, Rachel R, Sumper M (1997). Characterization of a 200-kDa diatom protein that is specifically associated with a silica-based substructure of the cell wall. Eur J Biochem.

[33] Hecky R, Mopper K, Kilham P, Degens E (1973). The amino acid and sugar composition of diatom cell-walls. Mar Biol.

[34] Naik RR, Brott LL, Clarson SJ, Stone MO (2002). Silica-precipitating peptides isolated from a combinatorial phage display peptide library. J Nanosci Nanotechnol.

[35] Muradov N, Taha M, Miranda AF, Wrede D, Kadali K, Gujar A (2015). Fungal-assisted algal flocculation: application in wastewater treatment and biofuel production. Biotechnol Biofuels.

[36] Powell RJ, Hill RT (2013). Rapid aggregation of biofuel-producing algae by the bacterium *Bacillus* sp. strain RP1137. Appl Environ Microbiol.

[37] Chungjatupornchai W, Fa-Aroonsawat S (2008). Biodegradation of organophosphate pesticide using recombinant cyanobacteria with surface- and intracellular-expressed organophosphorus hydrolase. J Microbiol Biotechnol.

[38] Chungjatupornchai W, Kamlangdee A, Fa-Aroonsawat S (2011). Display of organophosphorus hydrolase on the cyanobacterial cell surface using *Synechococcus* outer membrane protein a as an anchoring motif. Appl Biochem Biotechnol.

[39] Ferri S, Nakamura M, Ito A, Nakajima M, Abe K, Kojima K (2015). Efficient surface-display of autotransporter proteins in cyanobacteria. Algal Res.

[40] Wang SB, Hu Q, Sommerfeld M, Chen F (2004). Cell wall proteomics of the green alga *Haematococcus pluvialis* (Chlorophyceae). Proteomics.

[41] Guillard RR, Ryther JH (1962). Studies of marine planktonic diatoms. I. *Cyclotella nana* Hustedt, and *Detonula confervacea* (cleve) Gran. Can J Microbiol.

[42] Maeda Y, Sunaga Y, Yoshino T, Tanaka T (2014). Oleosome-associated protein of the oleaginous diatom *Fistulifera solaris* contains an endoplasmic reticulum-targeting signal sequence. Mar Drugs.

[43] Sunaga Y, Maeda Y, Yabuuchi T, Muto M, Yoshino T, Tanaka T (2015). Chloroplast-targeting protein expression in the oleaginous diatom *Fistulifera solaris* JPCC DA0580 toward metabolic engineering. J Biosci Bioeng.

[44] Nojima D, Yoshino T, Maeda Y, Tanaka M, Nemoto M, Tanaka T (2013). Proteomics analysis of oil body-associated proteins in the oleaginous diatom. J Proteome Res.

[45] Muto M, Fukuda Y, Nemoto M, Yoshino T, Matsunaga T, Tanaka T (2013). Establishment of a genetic transformation system for the marine pennate diatom *Fistulifera* sp. strain JPCC DA0580-a high triglyceride producer. Mar Biotechnol.

