# Supplementary material for: Peptide-mediated microalgae harvesting method for efficient biofuel production
Source: Biotechnol Biofuels. 2016 Jan 13;9:10. doi: 10.1186/s13068-015-0406-9 (PMC4712521; doi:10.1186/s13068-015-0406-9)
Supplement: Supplementary file 1 — 10.1186/s13068-015-0406-9 Supplementary Figures S1, S2, S3, S4, S5, S6, and S7. Supplementary Table S1. [file 13068_2015_406_MOESM1_ESM.pdf]

Supplementary information for:

Peptide-mediated microalgae harvesting method for efficient biofuel production

Yoshiaki Maeda[1], Takuma Tateishi[1], Yuta Niwa[1], Masaki Muto[1,2], Tomoko Yoshino[1], David Kisailus[3], Tsuyoshi Tanaka[1,2]

[1]Division of Biotechnology and Life Science, Institute of Engineering, Tokyo University of Agriculture and Technology, 2-24-16, Naka-cho, Koganei, Tokyo 184-8588, Japan
[2]JST, CREST, Sanbancho 5, Chiyoda-ku, Tokyo 102-0075, Japan
[3]Department of Chemical and Environmental Engineering, University of California, Riverside; Room 343, Materials Science and Engineering Building, Riverside, California 92521, United States

*Corresponding author
E-mail address: tsuyo@cc.tuat.ac.jp
Tel: +81-42-388-7401
Fax: +81-42-385-7713

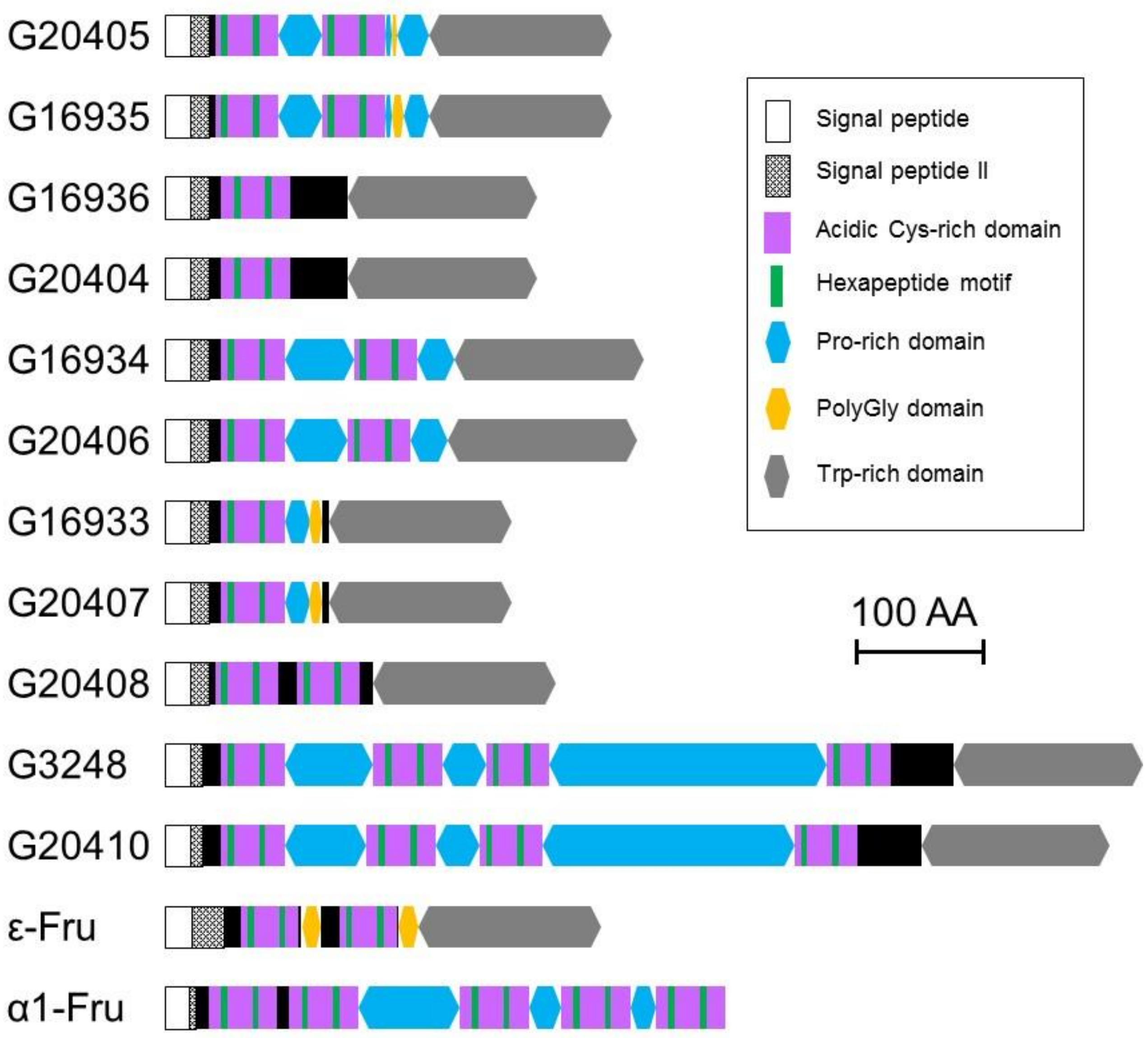

Fig. S1 Schematic of primary structures of frustulins found in the genome of *Fistulifera solaris* JPCC DA0580.

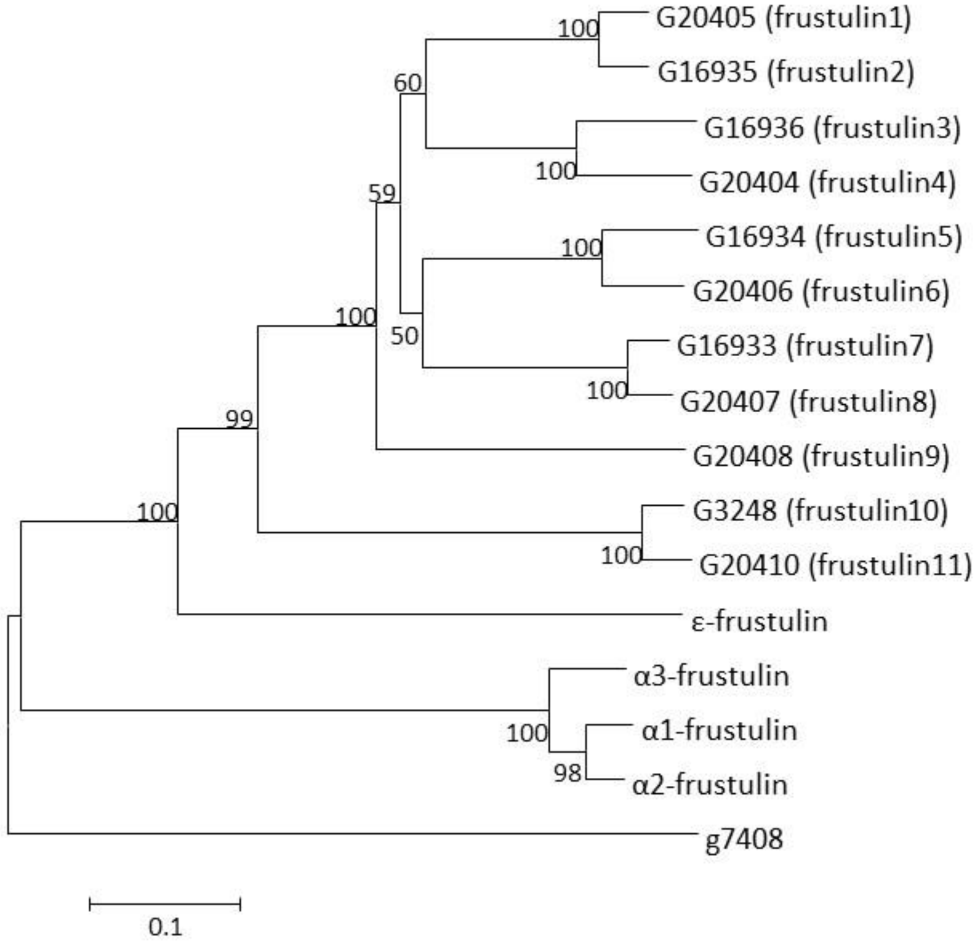

Fig. S2 Comparison of the amino acid sequences of frustulins identified from *F. solaris*, ε-frustulin from *Navicula pelliculosa* and α1-, α2-, α3-frustulin derived from *Cylindrotheca fusiformis*. The phylogenetic tree was constructed using the neighbor-joining method. Bootstrap trials were replicated 1000 times. Gaps were treated as missing data.

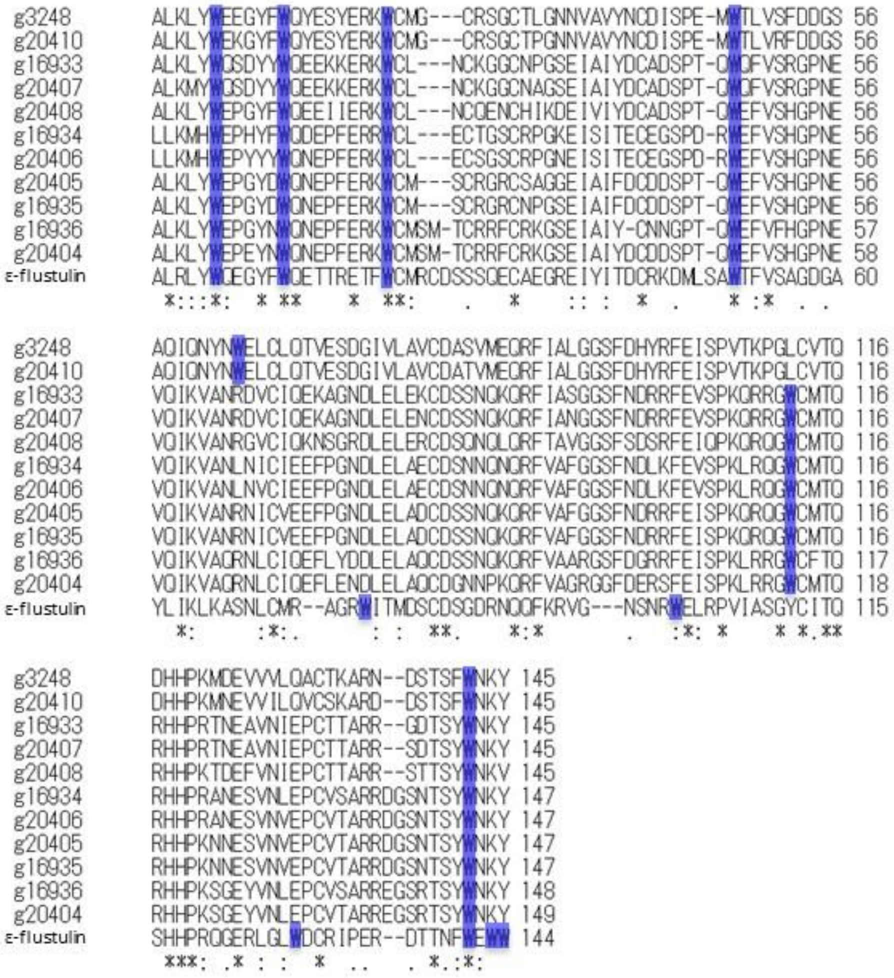

Fig. S3 Comparison of the amino acid sequences of the tryptophan-rich domains of frustulins identified from *F. solaris* and ε-frustulin from *Navicula pelliculosa.* ClustalW was used for the comparison. Characters highlighted in blue are tryptophan.

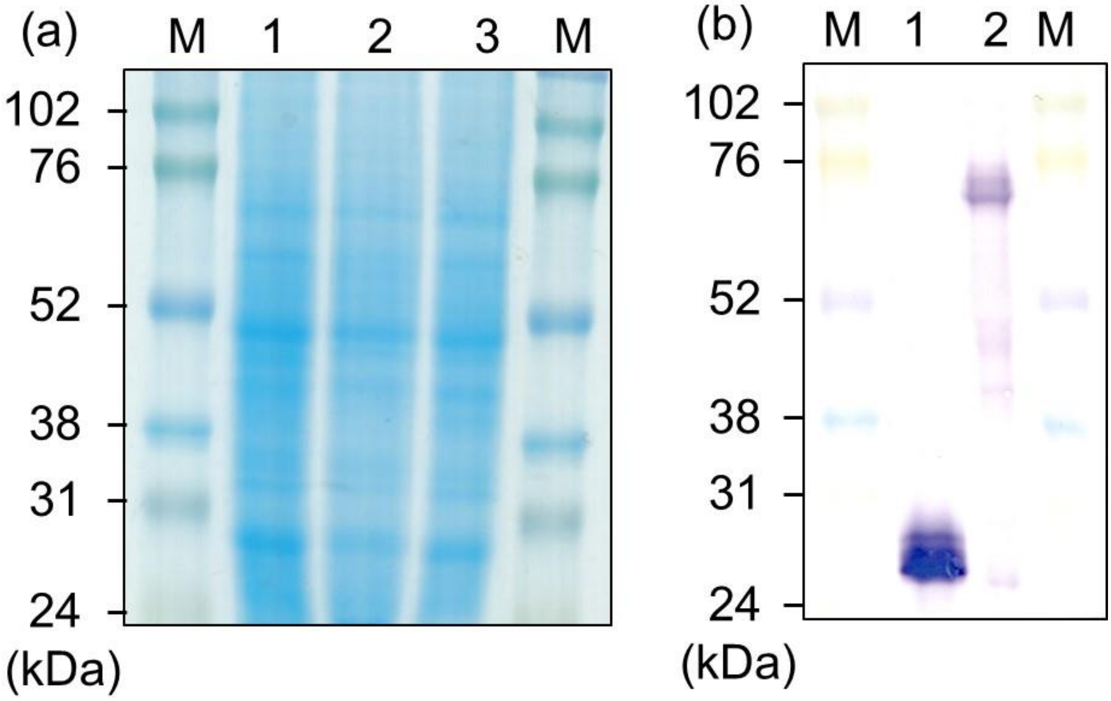

Fig. S4 SDS-PAGE and Western blotting analyses of the proteins extracted from the cells of *F. solaris* JPCC DA0580. (a) The proteins extracted from wild type (Lane 1), the transformant expressing GFP (Lane 2), and the transformant expressing frustulin1-GFP (Lane 3) with the SDS-boiling method were applied to the 10% polyacrylamide gel. It confirms that nearly equal amount of proteins were tested, but specific bands could not be found from transformants, which could be due to the low sensitivity of Coomassie brilliant blue staining. (b) The separated proteins by SDS-PAGE were then transferred to the membrane, and subjected to Western blotting with ALP-labeled anti-GFP antibody for more sensitive detection of GFP. GFP (Lane 1) and frustulin1-GFP were observed. M indicates molecular markers.

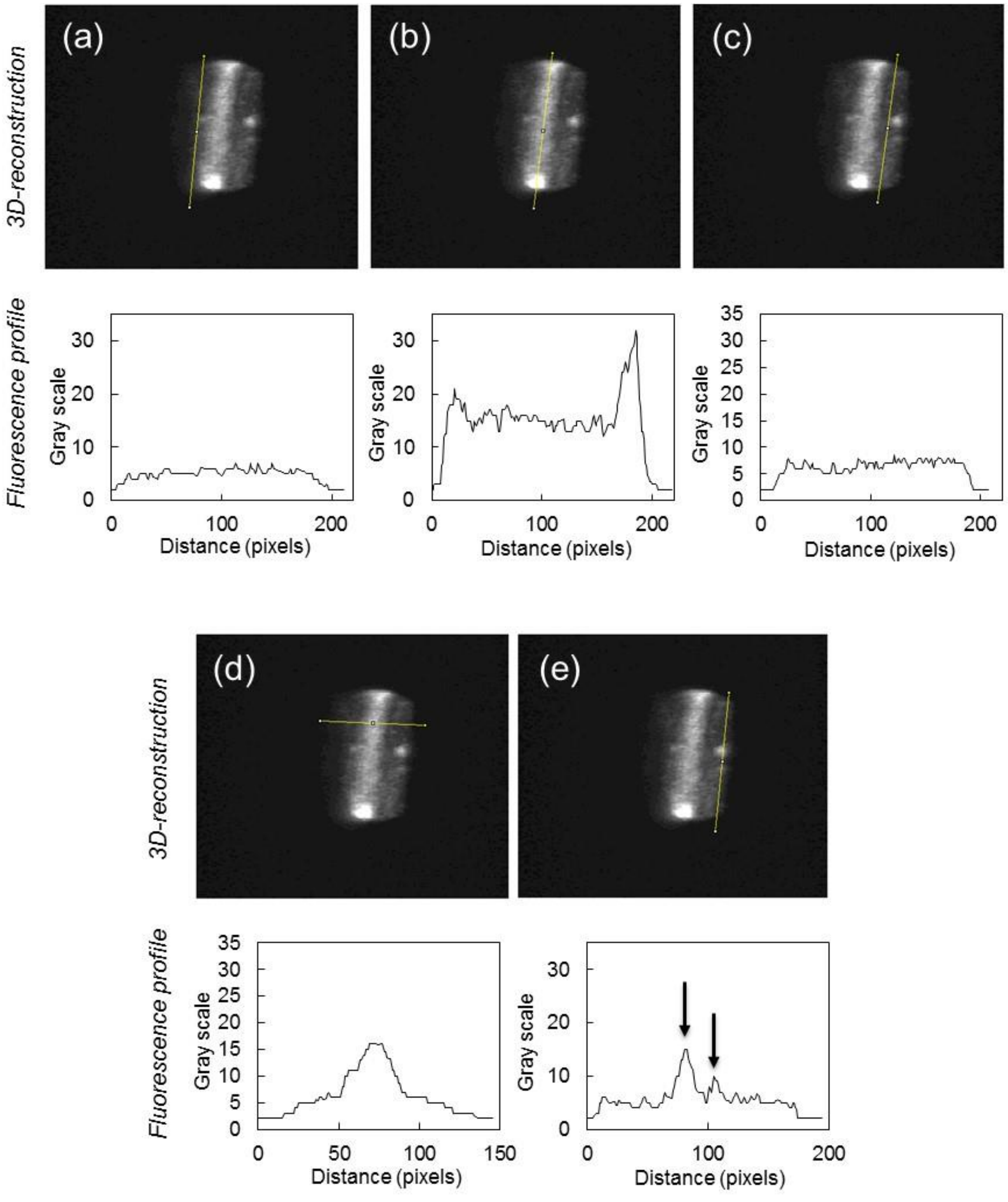

Fig. S5 Fluorescent signal profiling on the transformant cell expressing frustulin1-GFP. The profiling was performed along the valve regions (a, c), a central region of the girdle face (b), across the girdle face, and isolated points showing intensive signal (e, the isolated points were shown by arrows). Yellow lines are the profiled regions.

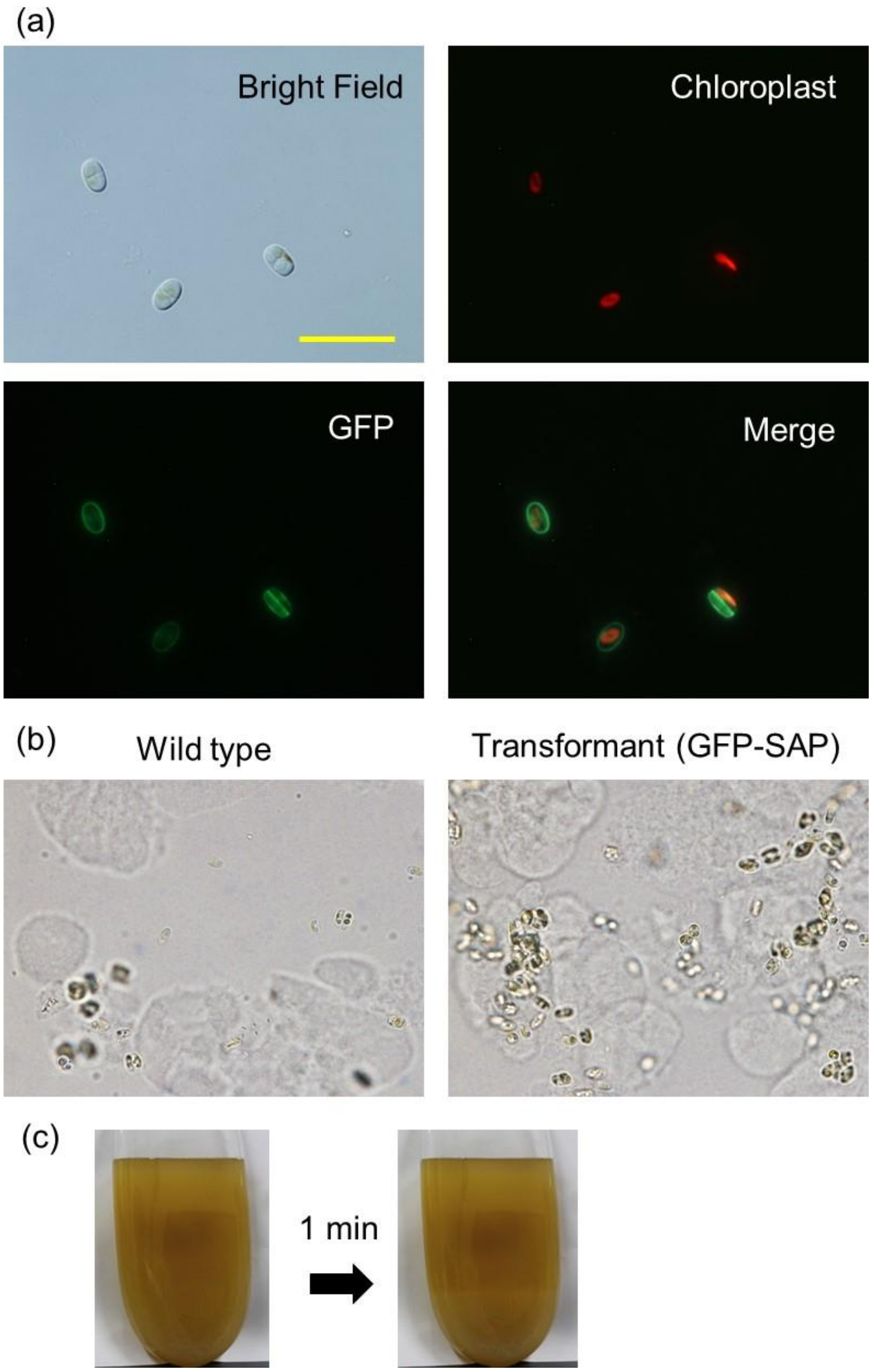

Fig. S6 SAP-mediated flocculation by co-cultivation of the diatom transformant and silica particles. (a) Bright field and fluorescent observations of the transformant cells expressing frustulin1-GFP-$(G_4S)_3$-SAP. (b) Precipitated silica particles (2.5 g) collected from the bottom of the flat-shaped flask containing the wild type or transformant cells displaying GFP-SAP cultures. (c) Silica particles were added after completing the transformant diatom cultivation, instead of co-cultivation.

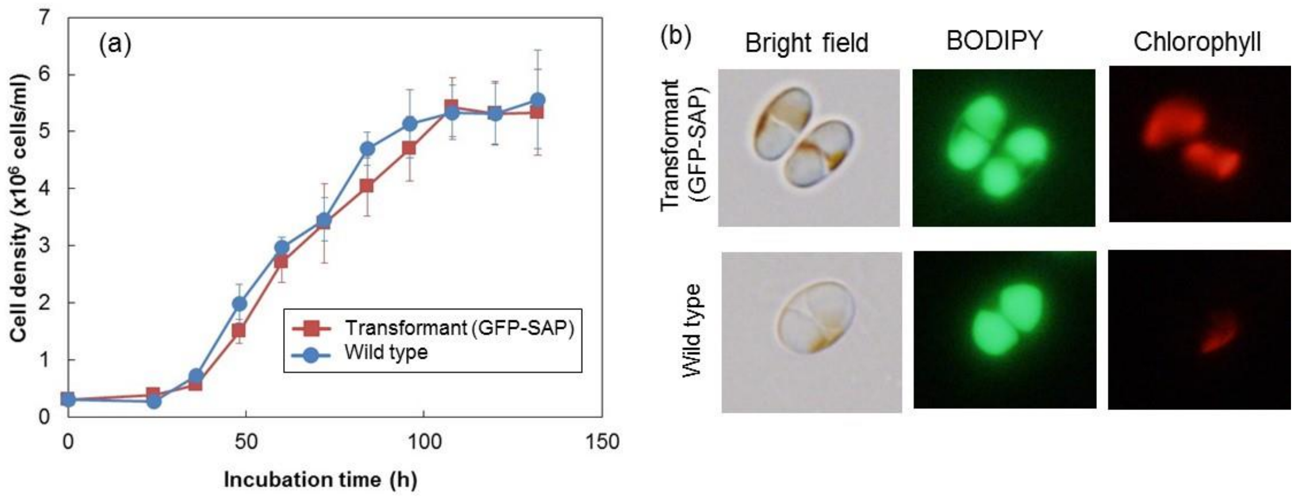

Fig. S7 Comparison of cell growth and lipid accumulation between the *F. solaris* wild type and transformant displaying GFP-SAP. (a) Cell growth comparison based on cell counting. (b) Lipid accumulation comparison based on BODIPY 505/515 (Molecular Probes; 25 mg/mL, 2% DMSO [v/v] in water) staining. BODIPY staining was performed by mixing cell culture and the dye (99:1 volume ratio) and incubated for 10 min at room temperature. The stained cells were observed by fluorescence microscopy (BX51; Olympus). Green labeled-oil bodies occupied almost of the area of both wild type and the transformant cells.

Table S1 Expression level of frustulins of *F. solaris* JPCC DA0580 incubated in the f/2 medium. Transcriptome analysis was performed using a Genome Analyzer IIx (Illumina) every 48 hours during the cultivation. RPKM values were calculated.

Gene ID	Name	48 hours	96 hours	144 hours
fso:g20405	Frustulin 1	67.69	54.61	54.96
fso:g16935	Frustulin 2	94.62	24.55	48.72
fso:g16936	Frustulin 3	0.07	5.69	19.49
fso:g20404	Frustulin 4	0.79	17.33	38.38
fso:g16934	Frustulin 5	25.27	12.83	12.43
fso:g20406	Frustulin 6	8.69	8.32	9.69
fso:g16933	Frustulin 7	14.05	4.88	11.75
fso:g20407	Frustulin 8	36.21	31.62	45.14
fso:g20408	Frustulin 9	3.37	64.94	45.87
fso:g3248	Frustulin 10	2.34	0.33	2.9
fso:g20410	Frustulin 11	2.22	0.27	1.56

Supplementary movie S1: Silica-affinity peptide (SAP)-mediated cell harvesting for the *F. solaris* transformant expressing frustulin1-GFP-$(G_4S)_3$-SAP. Culture media of the wild type (left) and transformant displaying GFP-SAP (right) co-incubated with silica particles (2.5 g). Co-flocculation of diatom transformant cells and silica particles were monitored.
